# A repetitive mutation and selection system for bacterial evolution to increase the specific affinity to pancreatic cancer cells

**DOI:** 10.1371/journal.pone.0198157

**Published:** 2018-05-31

**Authors:** Masaki Osawa

**Affiliations:** Department of Cell Biology, Duke University Medical Center, Durham, North Carolina, United States of America; CNR, ITALY

## Abstract

It is difficult to target and kill cancer cells. One possible approach is to mutate bacteria to enhance their binding to cancer cells. In the present study, Gram-negative *Escherichia coli* and Gram-positive *Bacillus subtilis* were randomly mutated, and then were positively and negatively selected for binding cancer vs normal cells. With repetitive mutation and selection both bacteria successfully evolved to increase affinity to the pancreatic cancer cell line (Mia PaCa-2) but not normal cells (HPDE: immortalized human pancreatic ductal epithelial cells). The mutant *E*. *coli* and *B*. *subtilis* strains bound to Mia PaCa-2 cells about 10 and 25 times more than to HPDE cells. The selected *E*. *coli* strain had mutations in biofilm-related genes and the regulatory region for a type I pilus gene. Consistent with type I pili involvement, mannose could inhibit the binding to cells. The results suggest that weak but specific binding is involved in the initial step of adhesion. To test their ability to kill Mia PaCa-2 cells, hemolysin was expressed in the mutant strain. The hemolysin released from the mutant strain was active and could kill Mia PaCa-2 cells. In the case of *B*. *subtilis*, the initial binding to the cells was a weak interaction of the leading pole of the motile bacteria. The frequency of this interaction to Mia PaCa-2 cells dramatically increased in the evolved mutant strain. This mutant strain could also specifically invade beneath Mia PaCa-2 cells and settle there. This type of mutation/selection strategy may be applicable to other combinations of cancer cells and bacterial species.

## Introduction

A potential approach to cancer treatment is to target the cancer cells with toxic reagents. A problem is that surface markers uniquely present on cancer cells are limited [1, 2]. The surface of cancer cells is actually considerably different from normal tissue cells. However, almost all differences, including protein expression, glycosylation, and lipid composition, are quantitative; the difference is not the species of molecules but their amounts [3–5]. One possibility to target cancer cells would be a probe that recognized the quantitative difference in surface epitopes, perhaps by interacting weakly with multiple types of molecules that are expressed more highly on cancer cells. The cooperativity of multiple weak interactions might result in increased specificity for binding to cancer cells.

Bacteria may be a good candidate for this type of tool because of their large surface area and many surface ligands including protein, carbohydrates and lipid that are available to develop interactions with cancer cells. One interesting observation is that various *B*. *subtilis* strains have largely different affinities for mucin, matrigel and a heterogeneous human epithelial colorectal adenocarcinoma cell line (Caco-2 cells) [6]. This suggests that random mutations might affect the bacterial surface and alter their binding to cell surface antigens. Therefore it may be expected that a simple mutation/selection system could create bacteria that have higher affinity to cancer cells.

There are several advantages to use bacteria to fight cancer. First, some bacteria have a natural capability to target cancer regions. Obligatory anaerobic bacteria such as *Bifidobacteria sp*. and *Clostridia sp*. prefer environments where there are no free oxygen molecules. Therefore we may expect that the hypoxic regions in tumors are a good home for them; in fact *C*. *novyi* has been found to concentrate throughout cancer regions [7]. This simple story, however, may not be universal because facultative anaerobic bacteria such as *E*. *coli* and *Salmonella*, which can live in both normal and hypoxic condition, can also accumulate in cancer regions in mice [8, 9]. There are some unknown mechanisms in cancer microenvironments which attract and/or support these bacterial growths.

Second, some bacteria naturally induce immunostimulation and attenuate cancer growth. One successful bacterial therapy for cancer has been to use BCG *(*Bacillus Calmette–Guérin) which is attenuated *Mycobacterium bovis*. Although the mechanisms are not fully understood, the main effects of BCG are to activate multiple immune pathways [10]. In the case of *E*. *coli*, not only was the tumor cleared in a mouse model through CD8(+) T cells, but also in re-challenging mice that had cleared tumors, new tumors were rejected through both memory CD8(+) and CD4(+) T cells[11]. Although these types of immunostimulation may be generalized with bacterial applications to various types of cancers, it has not yet translated into cancer treatments.

Third, bacteria can work as a vehicle for drug delivery. One approach is to enhance the immunostimulation by expressing and secreting artificial cytokines from engineered bacteria [12]. The simplest approach would be to release toxic molecules from bacteria to directly kill cancer cells. Secretion of a bacterial toxin such as alpha- hemolysin (aHL) and azurin have been shown to kill cancer cells in mice [13–15]. Bacteria that deliver a converting enzyme for prodrug have been able to reduce cancer mass in mice [16, 17].

Pancreatic ductal adenocarcinoma is one of the deadliest cancers, with 4% survival rate for 5 years. It is very difficult to target pancreatic ductal adenocarcinoma cells, and there is not even an effective biomarker for diagnosis. However, one interesting approach has been selection of random aptamers reported by cyclic negative and positive selection for binding to secreted materials from non-cancer pancreatic epithelial cells vs pancreatic ductal adenocarcinoma cells [18]. I have used a similar strategy in the present study. Instead of aptamers I have randomly mutated bacteria and applied negative and positive selection for binding to normal and cancer cells. The repetitive mutation/selection system produced bacteria that specifically bind to the pancreatic ductal adenocarcinoma cells.

## Methods

### Cells and bacterial strains

Immortalized human pancreatic ductal epithelial (HPDE) cells [19] and pancreas ductal adenocarcinoma cell lines (MIA PaCa2 cells) were kindly donated by Dr. M. S. Tsao, Ontario Cancer Institute and Dr. R. R. White, University of California San Diego. HPDE cells were cultured in Keratinocyte SFM with supplements and MIA PaCa2 cells were cultured in DMEM with 10% serum, unless otherwise specified. *E*. *coli* strain W3110 and *B*. *subtilis* strain 168 C were kindly donated by Dr. M. J. Kuhen, Duke University and Dr. J. Errington, Newcastel University, respectively. These bacteria were cultured in LB medium.

### Mutagenesis with UV irradiation

One ml samples of stationary cultured bacteria were frozen for stocks and thawed in 10 ml of LB. The suspension of bacteria was cultured at 37°C for 2 hrs for *E*. *coli* and 3 hrs for *B*. *subtilis*. One ml of bacteria was spun down and resuspended in 0.5 ml of 0.1 M MgSO_4_. The bacteria were placed on a plastic dish as 20 μl x 25 drops and exposed to UV until 99% of bacteria were killed. This has been reported to cover all possible single mutations in the genome [20]. UV treated bacteria were cultured in 5 ml LB overnight and stored as frozen stock.

### Negative and positive selection for mutated bacteria against cancer cells

**Fig 1:**
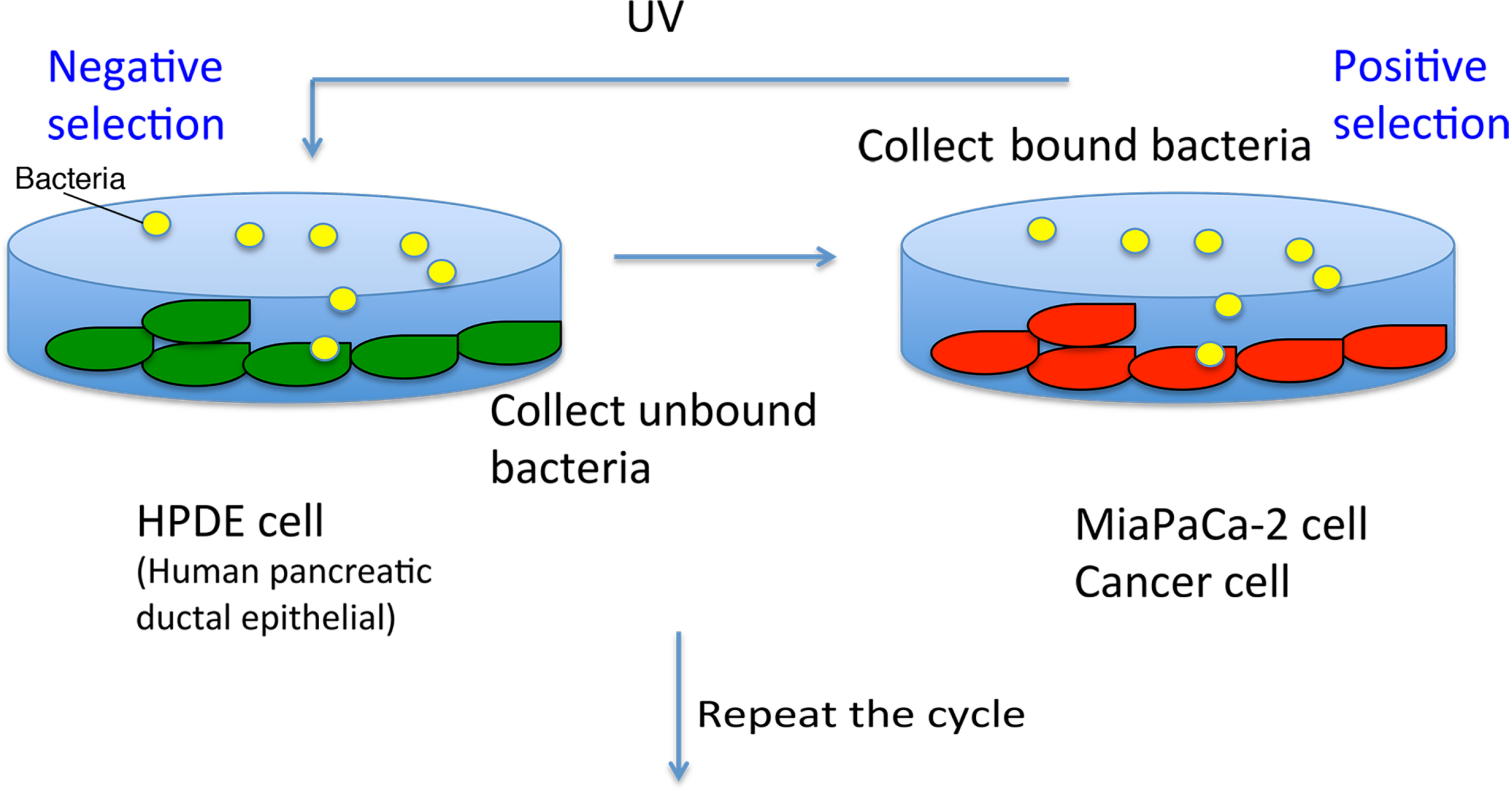
Repetitive mutation and selection system.

One ml of mutated bacteria was diluted 10 times in LB and cultured at 37°C for 2.5 hr for *E*. *coli* and 3.5 hr for *B*. *subtilis*. Typically 100 μl of bacteria was spun down and resuspended with conditioned media of HPDE cells. For negative selection HPDE cells were cultured as a confluent monolayer in T75 culture flasks (Corning) and just before addition of bacteria, the medium was removed except for 1 ml left in the flask. The resuspended bacteria (100 μl) were added to the HPDE cells. To spread bacteria on the entire cell surface, the flask was tilted several times and incubated at room temperature for 20 min with occasional tilting. The 1.1 ml supernatant, which contained the bacteria not bound to HPDE cells, was removed and transferred to positive selection system (Fig 1).

For positive selection, MIA PaCa-2 cells were cultured to confluence in a T 25 flask. The medium was removed except for 1 ml left in the flask. The negatively selected bacteria were added to the MIA PaCa-2 cells and incubated for 10 min with occasional tilting to spread the bacteria. Unbound bacteria were removed by aspiration and the MIA PaCa-2 cells were washed 10 times with 10 ml of PBS (Fig 1).

The bacteria not only bind to the confluent cultured MIA PaCa-2 cells but also bind to the plastic surface exposed at the sides and top of the flask. This inadvertent binding to the plastic surface mainly occurred during the washing with PBS. To kill the bacteria stuck to the plastic surface, after carefully removing the PBS the flask was placed upside down and 1 ml of 70% ethanol was added. Because the flask was upside down, the ethanol spread on the top surface of the flask and killed bacteria attached there. The ethanol was replaced with a fresh 1 ml of 70% ethanol, and the flask was tilted 90 degree in 3 directions for 1 s, and each time quickly put back to the upside down position. This process killed bacteria attached to the 3 sidewalls. With the flask still upside down, the ethanol was removed and the flask neck and exit were wiped with a swab soaked with 70% ethanol. Then 5 ml LB was added and flask was flipped upright. The bacteria stuck on the cells were cultured overnight in the flask. The cells were killed and detached from the flask as bacteria grow. The bacteria from this overnight culture were frozen and stored at -80°C. The frozen stocks were thawed and used for the next cycle of radiation.

### Bacterial adhesion assay

The bacteria were grown in LB overnight, then diluted 10 times with LB and cultured at 37°C for 2 h for *E*. *coli* and 3 h for *B*. *subtilis*. One ml of bacteria was spun down and resuspended to 0.1 ml PBS with 10 μg/ml FM4-64FX, a red fluorescent dye that stains the bacterial membranes. After 25 min incubation the bacteria were washed twice with 1ml PBS and resuspended in 1 ml conditioned medium from MIA PaCa-2 or HPDE cells. Fifty μl (for *E*. *coli*) or 150 μl (for *B*. *subtilis*) of the bacterial suspension (containing 0.3–0.7 x 10^8^ bacteria as assayed by colony formation) was diluted with 0.95 (for *E*. *coli*) or 0.85 ml (for *B*. *subtilis*) of conditioned medium and the adhesion assay was started by replacing culture medium of the cells with the diluted bacterial suspension. The bacteria were incubated with cells for 10 min which is relatively short compared to usual adhesion assays, to avoid complex cellular reactions and changing surface properties of the cells during a long incubation. With this condition, the mutant bacteria associated with cells in the range of one bacteria/cell, which was sufficient to show a statistically significant difference of binding for each strain. After 10 min incubation, the cells were washed with 1 ml PBS three times and subsequently fixed with 4% formaldehyde for 10 min. The fixed cells were washed 4 times with 1 ml PBS and stored at 4°C. At least 100 pancreatic cells were observed for each experiment to count the adherent bacteria.

The optimal culture media were different for MIA PaCa-2 and HPDE cells. To test whether culture media affected the adhesion, some assays were conducted with the cells in identical medium. In this case cells were plated on a glass surface coated with a fibronectin fragment FN7-10 containing the RGD peptide [21], and grown in Keratinocyte SFM with supplements.

### Genomic PCR

To estimate the change in FimB/E activity in mutant *E*. *coli* (ECUV10c), *fimS* direction was checked by genomic PCR. The genomes from wild type (wt) *E*. *coli* (W3110) and mutant *E*. *coli* (ECUV10c) were purified by a genome purification kit (quick gDNA, Zymo research). The forward primer “ggcatatcggcatgggatgcgtatta” was in the *fimE* gene that is located before *fimS*. The reverse primer for the active direction for *fimS* (the active direction for the promoter of *fimA*) is atgcgtcgagccacagaaacgttagc, which amplifies a 710 bp product. The reverse complement of this primer was used for the inactive direction for fimS and the product size is 718 bp. The important measure is the intensity of the bands for the ON and OFF primers. The PCR condition was carefully adjusted to the linear range of a standard curve so that the results could be fitted for relative quantifications between wt *E*. *coli* and mutant.

### aHL expression and killing Mia PaCa-2 cells

The aHL gene was purchased as a double strand DNA fragment (gblock, integrated DNA Technologies) with flanking EcoRI–HindIII sites. The aHL gene was spliced into three kinds of pBAD based plasmids for expression in *E*. *coli*. The first construct was pBAD18 [22], which is referred to as pBAD_18aHL. The second construct improved the Shine-Dalgarno region of pBAD18_aHL. The sequence ctagcgaattcATG (start codon) was replaced with ctaacaggaggaattaaccATG (start codon) by doing PCR for the entire plasmid followed by ligation. This plasmid is referred to as pBADMO_aHL, and is expected to give a higher expression level of aHL. To make the third plasmid, the araE gene with Pcp8, which is a constitutively active promoter, was obtained by PCR from the genomic DNA of BW27783 strain [23] and was inserted between the M13 intergenic region and pBR322 ori in pBADMO_aHL by creating AscI–NcoI site in both pBADMO_aHL and Pcp8-araE. This plasmid is referred to as pBADMOE_aHL, and is expected to give a more uniform level of expression of aHL from cell to cell [23].

The mutant *E*. *coli* strain ECUV10c (cloned mutant *E*. *coli* after treatment with 10 cycles of UV irradiation/selection) was transformed with each of the three plasmids and single colonies were cultured overnight in LB. For aHL induction, the cultures were diluted 20 or 100 times in LB with 0.2% arabinose grown in a shaker at 37°C for 4 h or overnight. After induction, *E*. *coli* was spun down and the supernatant, containing aHL released into the medium, was collected. The aHL was concentrated 40 times by a centrifugal filter with a 10 kDa cut-off (Sigma). This aHL was applied to Mia PaCa-2 cells cultured in bottom glass dishes (Mattech), after dilution to 560 times in Mia PaCa-2 culture media, giving a 14-fold dilution from that in the original culture supernatant.

### Microscopy

Differential interference contrast and fluorescence images of bacteria and cells were obtained with a Zeiss Axiophot microscope with 100x (NA 1.3) or 40x (NA 1.3) objective lens and a CCD camera (CoolSNAP HQ, Roper). The FM-64 signal was captured through a rhodamine filter cube (Chroma BP546 FT580 BP590).

## Results

### Repetitive mutation/selection system

I took a simple strategy to obtain bacterial mutants that specifically bind to cancer cells. Bacteria randomly mutated with UV irradiation were subjected to negative selection with immortalized human pancreatic ductal epithelial (HPDE) cells and then positive selection with a pancreas ductal adenocarcinoma cell lines (MIA PaCa2 cells) (Fig 1 and see materials and methods). To concentrate bacteria that specifically bind to the cancer cells, three rounds of negative and positive selection without UV irradiation were applied after each UV irradiation (Fig 1). In many cases a reduced number of bacteria was applied for these additional selections to effectively concentrate the bacteria that have affinity to cancer cells. In general, 10^8^ bacteria were input to the negative/positive selection after UV irradiation, then 10^7^, 10^6^, and 10^5^ bacteria were input for second, third and fourth cycles of selection without UV irradiation. These numbers of bacteria should retain significant genomic variation.

These complete selection processes were repeated 10 and 9 times for two bacterial species, *E*. *coli* and *B*. *subtilis*. I chose these bacteria because their surfaces are very different; the Gram-negative bacteria have an outer membrane, while Gram-positive bacteria do not. This should give general insights into the mechanisms by which bacteria might increase their affinity to cancer cells by repetitive mutation/selection.

### Selected *E*. *coli* could specifically bind to Mia Paca-2 cells

**Fig 2:**
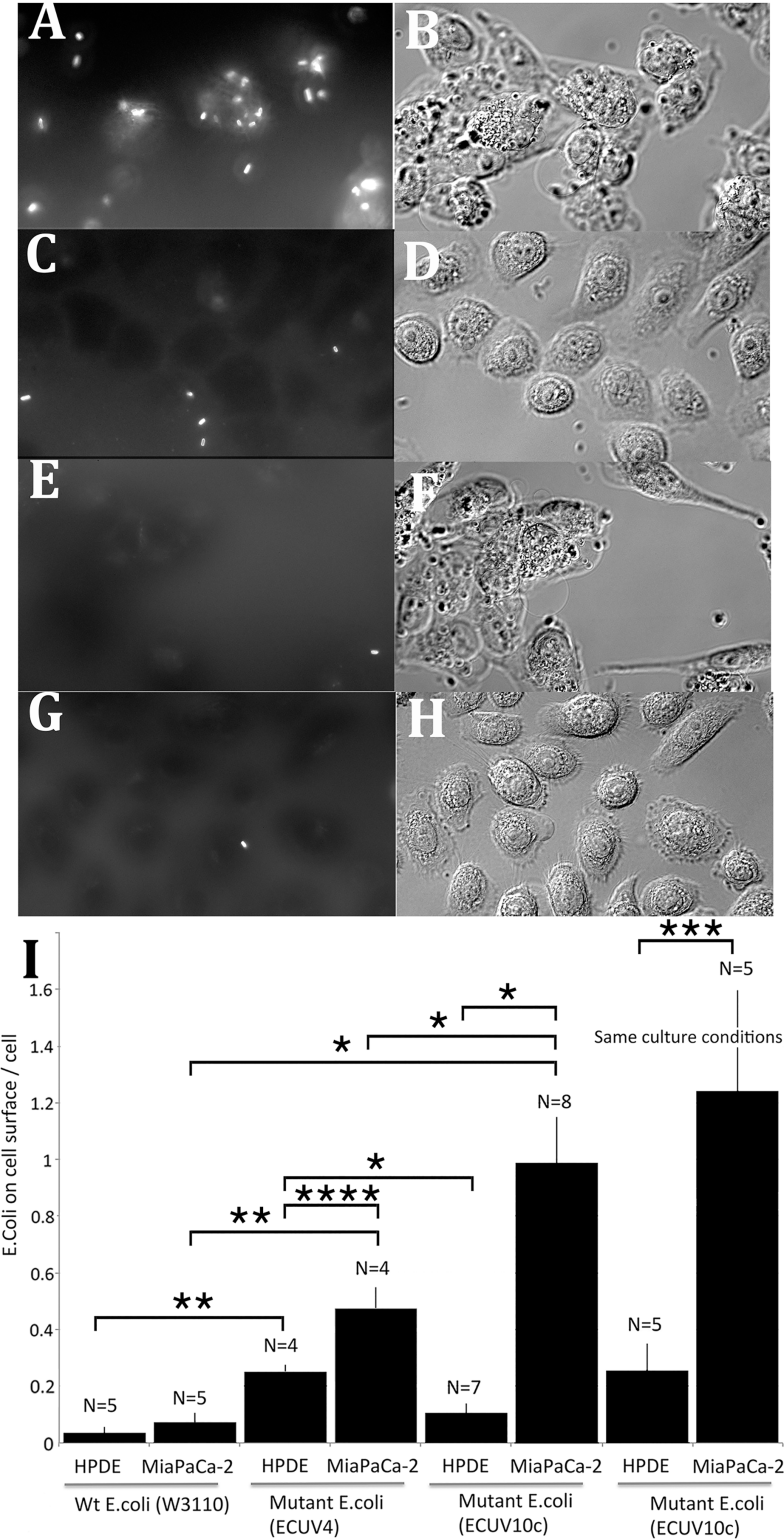
Mutant *E*. *coli* ECUV10c shows enhanced adhesion to pancreatic cancer cells. (A, B) Adhesion assay for ECUV10c with Mia PaCa-2 cells. (C, D) Adhesion assay for ECUV10c with HPDE cells. (E, F) Adhesion assay for wt *E*. *coli* with Mia PaCa-2 cells. (G, H) Adhesion assay for wt E. coli with HPDE cells. (A, C, E, G) show the fluorescently stained bacteria, and (B, D, F, H) show DIC images of the cells. (A, C) are superimposed images from two focal plane to show the maximum number of *E*. *coli* on the surface of the same cells in each DIC image (B, D). (I) Quantification of the adhesion assays. Approximately 0.5 x 10^8^ bacteria were added to the cells. The exact number confirmed by colony assay was 0.3–0.7 x 10^8^. The number of bacteria was normalized to 0.5 x 10^8^. The number of attached bacteria adhered to all focal levels of the cells was counted and averaged per cell. The intermediate mutant strain ECUV4 showed increased adhesion to both HPDE and Mia PaCa-2 cells. The further selection to ECUV10c decreased adhesion to HPDE and increased adhesion to Mia PaCa-2 cells. The last two columns show adhesion to HPDE and Mia PaCa-2 cells plated on a glass surface coated with FN7-10 and grown in Keratinocyte SFM medium. N shows number of independent experiments. Error bars are standard deviations. *, **, *** and **** show student t test P<0.0001, P<0.0005. P<0.001, and P<0.01, respectively.

To evaluate the bacterial adhesion to cancer cells, selected strains of both *E*. *coli* and *B*. *subtilis* were stained with the membrane dye FM4-64 and were applied to cells for 10 min as shown in Fig 2. The mutant *E*. *coli* (ECUV10c: cloned mutant *E*. *coli* treated with10 cycles of UV irradiation/selection) showed the highest attachment to Mia PaCa-2 cells, which was about 10 times more than to HPDE cells (Fig 2). In contrast, the original wt *E*. *coli* gave the lowest attachment to both Mia PaCa-2 cells and HPDE cells (Fig 2E, 2F, 2G, 2H and 2I). These results suggest that this mutation/selection system actually functions and successfully selected for mutant *E*. *coli* that specifically bind to the cancer cells. Interestingly, the intermediate mutant strain (ECUV4: heterogeneous mutant *E*. *coli* treated with 4 cycles of mutation/selection) increased the adhesion to both HPDE and Mia PaCa-2 cells compared to wt *E*. *coli*). Further cycles of mutation/selection to ECUV10c decreased adhesion to HPDE and increased adhesion to Mia PaCa-2 (Fig 2I). These results suggest that both positive and negative selections are functioning well.

One concern was that HPDE cells were cultured in Keratinocyte SFM with supplements, and Mia PaCa-2 cells were cultured in DMEM/10% serum. To circumvent this difference, I attempted to culture Mia PaCa-2 cells in Keratinocyte SFM and found that they did not adhere to glass (or to cell culture plastic) when they were plated. This may suggest that Mia PaCa-2 cells do not produce enough fibronectin by themselves. Therefore I coated the glass surface with fibronectin fragment FN7-10, which includes the RGD peptide for integrin binding [21]. Mia PaCa-2 cells could adhere and spread on this substrate in Keratinocyte SFM and grow with very similar morphology to the Mia PaCa-2 cells in DMEM/10% serum. Under this condition, the ECUV10c could attach to the Mia Paca-2 cells at essentially the same level as the Mia Paca-2 cells cultured in DMEM/10% serum (Fig 2I). Adhesion to HPDE was increased somewhat, but a large differential was maintained under these identical culture conditions.

The complete genomes of our laboratory wt W3110 strain and the mutant (ECUV10c) were determined by Illumina sequencing. The differences from the database W3110 strain (accession # PRJNA16351) are shown in Table 1. Our laboratory wt W3110 strain, the parent of ECUV10c, had 9 mutations from the database strain, including a mutation in *fliI*, the flagellum-specific ATP synthase. This is in agreement with the motility defect of laboratory strain W3110. This strain acquired 25 additional point mutations and an obvious large deletion during 10 cycles of UV irradiation with positive/negative selection. The large deletion corresponds to the e14 which is a UV-excisable defective prophage with 19 genes and 3 pseudogenes, *ymfO'*, *ymfP'* and *stfE'*, located at 1197893–1212987 in the database W3110 genome. This is reasonable because this strain received UV irradiation. Eight of 25 mutations were in the *icd* (isocitrate dehydrogenase) gene right next to e14 deletion. Notably all these 8 mutations in *icd* are silent mutations.

**Table 1:** Genomic mutations in the lab W3110 strain and ECUV10c.

POS	Type	W3110 REF	Lab strain W3110	ECUV10c	Gene	Protein mutaion	Function
547694	SNP	A	G	G	YlbE	E83E	hypothetical protein
547831	INDEL	A	AG	AG	YlbE	K86E flameshift	hypothetical protein
556858	SNP	A	T	T	folD	L36Q	(a)
926691	SNP	G	REF	A	infA	P59S	translation initiation factor IF-1
926692	SNP	G	REF	A	infA	T58T	translation initiation factor IF-1
928602	SNP	A	REF	T	cydC	L339Q	ATP binding/permease protein
1065700	SNP	C	T	T	yccE	R415C	hypothetical protein
1088940	SNP	C	REF	T	ycdR	G447D	(b)
1093686	SNP	T	C	C	ycdT	V150A	(c)
1182294	SNP	A	REF	T	ycfZ	L184STOP	predicted inner memb. protein
1197797	SNP	C	REF	T	icd	H366H	isocitrate dehydrogenase
1197809	SNP	C	REF	T	icd	T370T	isocitrate dehydrogenase
1197822	SNP	T	REF	C	icd	L375L	isocitrate dehydrogenase
1197824	SNP	A	REF	G	icd	L375L	isocitrate dehydrogenase
1197854	SNP	C	REF	T	icd	N385N	isocitrate dehydrogenase
1197857	SNP	G	REF	C	icd	A386A	isocitrate dehydrogenase
1197860	SNP	A	REF	G	icd	K387K	isocitrate dehydrogenase
1197869	SNP	C	REF	T	icd	T390T	isocitrate dehydrogenase
1197881	SNP	G	REF	A	icd	E394E	isocitrate dehydrogenase
1303712	SNP	A	G	G	oppA	S273G	oligopeptide transporter
1317208	SNP	C	REF	T	yciF	E93K	hypothetical protein
1624961	SNP	C	REF	A	ydeH	V202F	(d)
1646060	SNP	G	REF	A	intergenic		
2019202	SNP	C	T	T	fliI	T171I	flagellum ATP synthase
2627746	SNP	C	REF	T	intergenic		
2906975	SNP	C	G	G	pyrG	D450H	CTP synthetase
3449836	SNP	A	REF	C	rpoC	I499S	(e)
3735150	SNP	G	REF	A	bglH	V230V	(f)
3735183	SNP	G	REF	T	bglH	Q241H	(f)
4371270	INDEL	GAA	G	G	intergenic		
4421323	SNP	T	REF	A	yjfO	K25STOP	hypothetical memb. protein
4542607	INDEL	GC	REF	G	yjhT	A277 frameshift	hypothetical protein
4545485	SNP	G	REF	T	intergenic (fimB)		fimB promoter P1
4547053	SNP	C	REF	T	fimE	R115C	(g)

POS is the position in the W3110 genomic sequence (NCBI Reference Sequence: NC_000964.3). REF is the W3110 sequence. Lab W3110 strain has 9 mutations in the genome, compared to the database W3110 sequence.

(a) Bifunctional enzyme 5,10-methylene-tetrahydrofolate dehydrogenase and 5,10-methylene-tetrahydrofolate cyclohydrolase.

(b) Predicted enzyme associated with biofilm formation.

(c) Predicted diguanylate cyclase.

(d) conserved hypothetical protein diguanylate cyclase involved in biofilm formation.

(e) RNA polymerase beta prime subunit.

(f) carbohydrate-specific outer membrane porin (cryptic).

(g) tyrosine recombinase/inversion of on/off regulator of fimA

**Fig 3:**
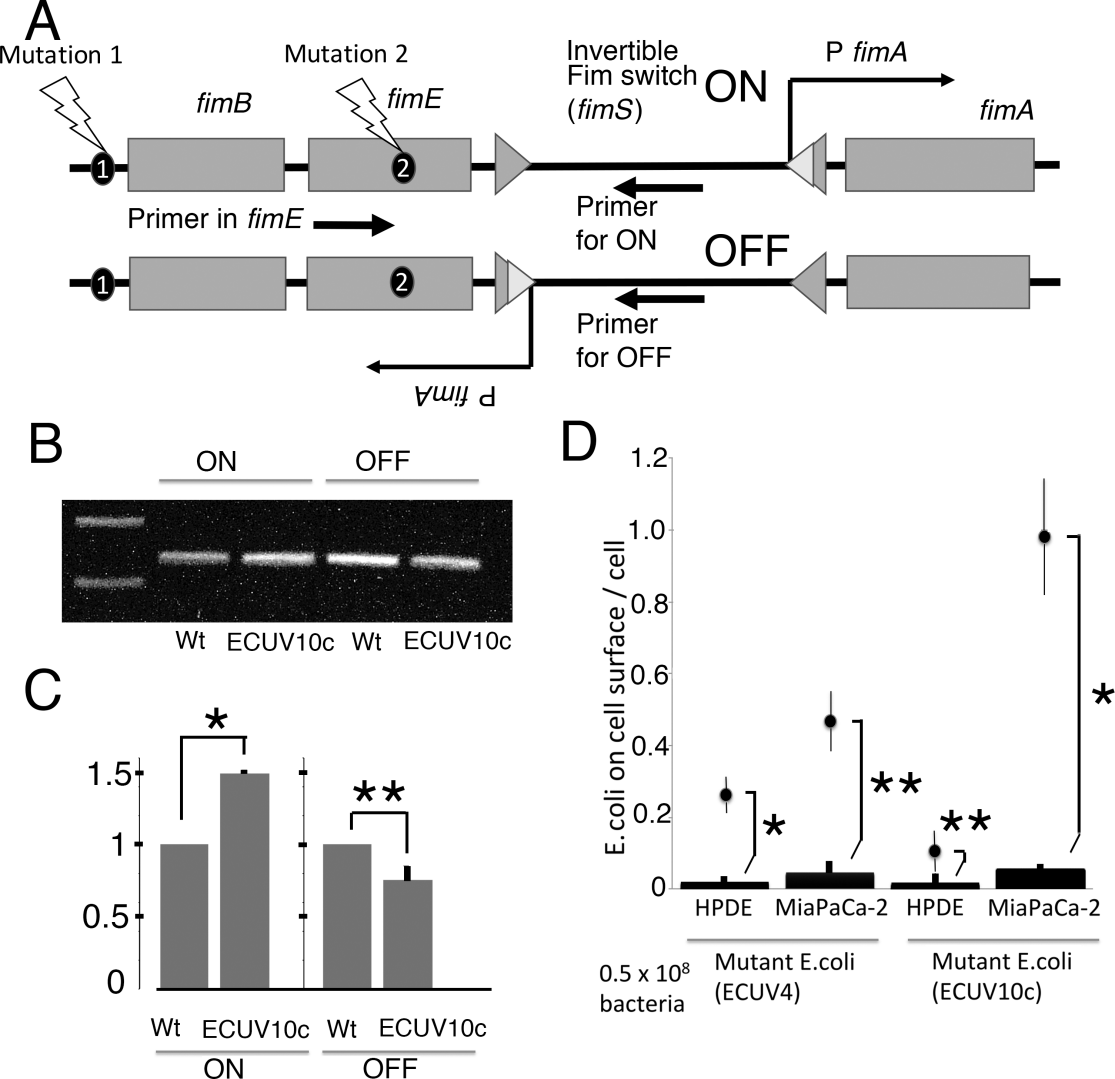
Fim switch (*fimS*) orientation and mannose inhibition of mutant *E*. *coli* adhesion to cells. (A) Schematic view of gene structure for *fimB*, *fimE*, *fimS*, and *fimA*. Mutation sites are shown as 1 and 2. The upper structure has the active orientation of *fimS* with *fimA* promoter that can express type I pili (Fim switch ON). The lower structure has an inactive orientation of *fimS* that cannot express type I pili (Fim switch OFF). Arrows show locations of primers for genomic PCR. Note that the entire *fimS* segment is inverted between ON and OFF. (B) Gel profile of genomic PCR products. The very left lane is the marker showing 1 and 0.5 kbp bands from the top. (C) quantification of band intensities. The values of PCR products from ECUV10c were normalized to those of wt. Four independent experiments were performed and error bars are standard deviations. * and ** show student t test P = 0.0001 and P = 0.016, respectively. (D) The adhesion assays were applied to ECUV4 and ECUV10c in the presence of 1% mannose. Adhesion in mannose is indicated by the bars at the bottom. Dots with error bars are replotted from Fig 2 (without mannose). More than four independent experiments for each combination were performed and error bars are standard deviations. * and ** show student t test P<0.0001 and P<0.0005, respectively, between the bar (with mannose) and the point (without mannose).

Three of the mutations are in the genes involved in biofilm formation (*ycdR*, *ydeH*, *yjfO*). These may affect the binding of ECUV10c to cancer cells by changing the surface of the bacteria. Probably the most important mutations are in the *fim* genes encoding type I pili, which have affinity to mannose residues. The mutations are located at 3 nucleotides before the transcription start of *fimB* gene, and in the ORF of the *fimE* gene (Fig 3A). Both FimB and FimE are recombinases that regulate the expression level of type I pili by flipping the promoter region of *fimA* which is the axis of the type I pili.

### Involvement of type I pili in *E*. *coli* adhesions

To check the FimB and FimE activity in ECUV10c, the *fimS* direction was estimated by genomic PCR (Fig 3B and 3C). The active direction of *fimS* that can express type I pili is significantly higher in ECUV10c than in wt. This difference was confirmed by the evaluation of the inactive direction of *fimS* which is lower in ECUV10c than in wt. This suggests that expression of type I pili increases in ECUV10c.

To check the involvement of type I pili for the binding of *E*. *coli* to the cancer cells, 1% mannose was added to the medium during the adhesion assay (mannose is known to block binding of type I pili to carbohydrate ligands). The adhesions of both ECUV4 and ECUV10c were greatly reduced relative to wt, suggesting that the adhesions are highly dependent on the type I pili (Fig 3D). However, these type I pili-dependent adhesions were probably regulated by additional factors. In the case of ECUV4, the type I pili-dependent adhesions for Mia PaCa-2 and HPDE cells were increased by the mutation/selection system (Figs 2 and 3D). Because there are no FimB/E mutations in this ECUV4 population (S1 Table), some other factors must enhance these adhesions to both Mia PaCa-2 and HPDE cells. The mutation in the *ycdR* gene may be responsible for this adhesion enhancement because about half of population in ECUV4 has a mutation in this gene (S1A Fig).

The ECUV10c strain has mutations in both *fimB* and *fimE* regions and probably has an increased number of the type I pili, which may increase the type I pili-dependent adhesions on Mia PaCa-2 cells (Fig 2). However, the same type I pili-dependent adhesions on HPDE cells were actually reduced for ECUV10c compared to ECUV4, again suggesting that there are some additional factors involved (see discussion).

### Killing Mia PaCa-2 cells with alpha-hemolysin released from ECUV10c

Previous studies have reported that *Staphylococcus aureus* alpha-hemolysin (aHL) kills cancer cells both in cell culture and in mice [13, 14]. However, this method can have problems because the sensitivity to aHL varies for different cancer cell lines, and also aHL lyses the expressing bacteria in some conditions [13, 14, 24]. To address these problems, I constructed three arabinose-inducible pBAD-based plasmids, which should give different expression levels of aHL. The supernatant of the *E*. *coli* expression cultures was tested for aHL by adding to culture medium of Mia PaCa-2 cells. When aHL was expressed from pBAD18_aHL in ECUV10c, the supernatant did not kill Mia PaCa-2 cells.

To increase the expression level of aHL, I made a second plasmid, pBADMO_aHL, which has an improved ribosomal binding site from pBAD18_aHL. The supernatant medium from a 4 h induction of ECUV10 with pBADMO_aHL could kill Mia PaCa-2 cells. The supernatant was concentrated 40 times with a centrifugal filter and diluted 560 times in the culture medium of Mia PaCa-2 cells, resulting in 14 fold dilution from the original supernatant.

To further enhance the expression level, a third plasmid, pBADMOE_aHL, was created. For this I incorporated into pBADMO_aHL an additional gene expressing AraE with a constitutively active promoter. It is known that expression of AraE (low affinity high capacity arabinose transporter) increases the protein expression level from pBAD, and also generates a more uniform response of individual bacteria. When aHL was expressed for 4 hrs from pBADMOE_aHL in ECUV10c, the supernatant medium, Mia PaCa-2 cells were killed at four times dilution from the concentration of the original supernatant. However, higher dilution did not have activity to kill Mia PaCa-2 cells, indicating that the aHL concentration from pBADMOE_aHL is lower than that from pBADMO_aHL. The reason for this is that ECUV10c with pBADMOE_aHL did not grow well in induction media, perhaps because the higher and uniform expression of aHL lysed ECUV10c, consistent with the previous report (S2 Fig) [13]. In contrast, ECUV10c with pBADMO_aHL could apparently grow while it released aHL into the media. As a result, the aHL activity (concentration) is higher in the media for ECUV10c with pBADMO_aHL than with pBADMOE_aHL.

**Fig 4:**
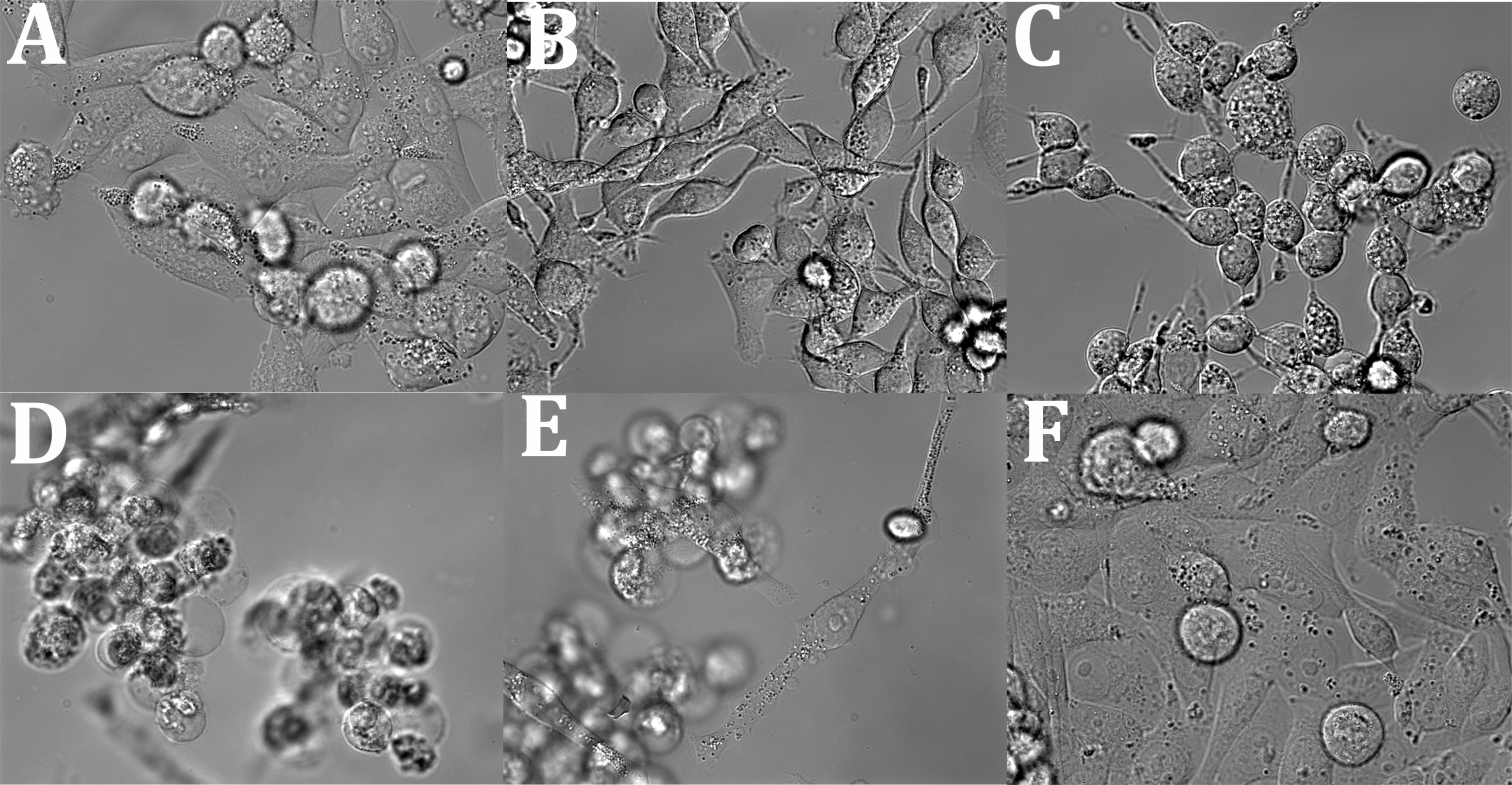
Killing Mia PaCa-2 cells by hemolysin released from ECUV10c. ECUV10c with pBADMO_aHL was induced for hemolysin expression with 0.2% arabinose. The supernatant of the culture was collected after 4h induction. 1.8 μl of concentrated supernatant was added to Mia PaCa-2 cells in 1ml of culture media, which corresponds to 14 times dilution from original ECUV10c supernatant. The images show cells (A) before addition of supernatant, (B) 1 h, (C) 4 h, and (D, E) overnight after addition. (F) is negative control with a Mock plasmid.

Mia PaCa-2 cells remained attached to the glass surface 1 h after addition of supernatant of ECUV10c with pBADMO_aHL, but they started shrinking and some of them had distinctive bubble like structures (Fig 4B). After 4 hr most cells were rounded up (Fig 4C). After overnight treatment almost all cells were detached from glass surface (Fig 4D and 4E).

### Selected *B*. *subtilis* can specifically bind to Mia PaCa-2 cells

**Fig 5:**
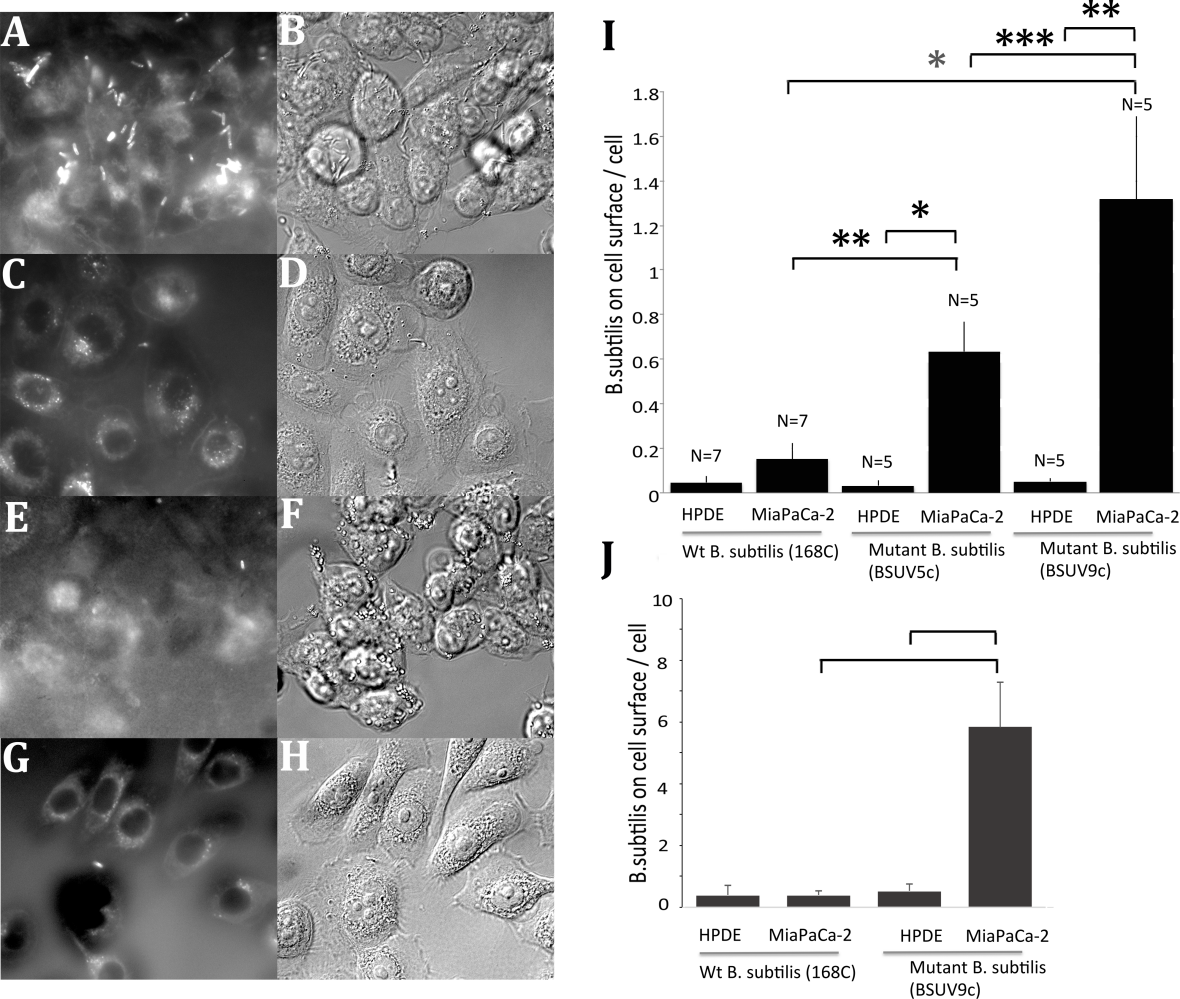
Increase of mutant *B*. *subtilis* adhesion to pancreatic cancer cells. (A, B) Adhesion assay for BSUV9c with Mia PaCa-2 cells. (C, D) Adhesion assay for BSUV9c with HPDE cells. (E, F) Adhesion assay for wt *B*. *subtilis* with Mia PaCa-2 cells. (G, H) Adhesion assay for wt *B*. *subtilis* with HPDE cells. Fluorescence images of FM4-64FX-stained bacteria are in (A, C, E, G) and DIC images of cells are in (B, D, F, H). (I) Quantification of the adhesion assays. The quantification was performed as described in Methods and Fig 2. N shows number of independent experiments. Error bars are standard deviations. *, **, and *** show student t test P<0.001, P<0.005 and P<0.01, respectively. (J) Quantification of attachment of bacteria without washing was determined from movies. Four independent movies were assayed for each point. Two combinations show P<0.005.

The same selection process and adhesion assays were applied to *B*. *subtilis*. FM4-64 could stain *B*. *subtilis* very well as shown in Fig 5. Although some transfer of the dye from attached *B*. *subtilis* to the cell membrane was observed, *B*. *subtilis* on cells were easily detected by fluorescence and confirmed in DIC images. Unlike *E*. *coli*, many mutant *B*. *subtilis* were found beneath cells probably because of their active motility. BSUV9c, the cloned mutant *B*. *subtilis* with 9 cycles of UV irradiation/selection, attached to Mia PaCa-2 cells 25 times more than to HPDE cells (Fig 5A, 5B, 5C, 5D and 5I). In contrast, the original wt *B*. *subtilis* attached to HPDE cells at about the same level as BSUV9c. The mutations in this case resulted in a large increase in attachment to Mia PaCa-2.

**Fig 6:**
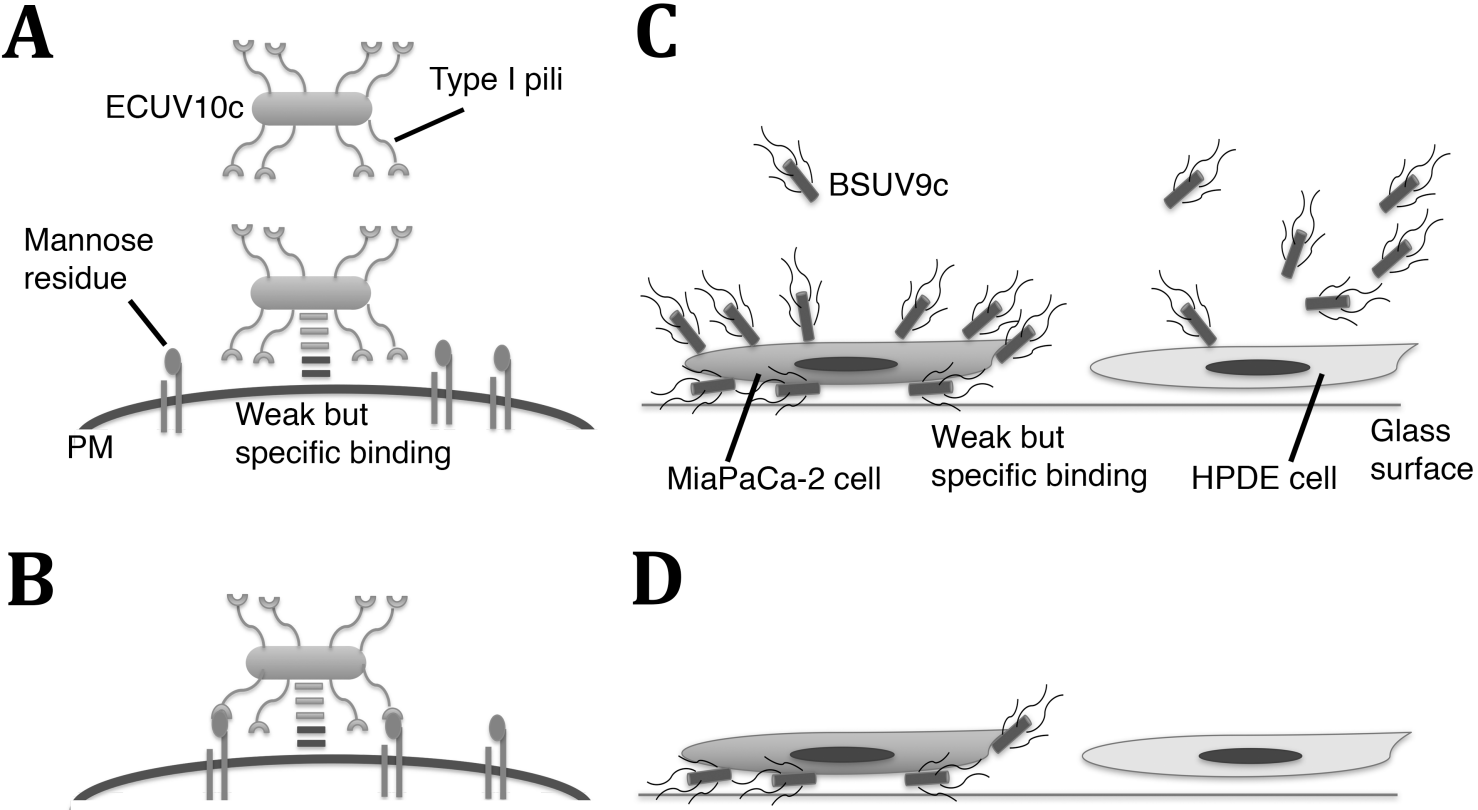
Models for how ECUV10c and BSUV9a may bind to the Mia PaCa-2 cells. (A) ECUV10c specifically binds to the Mia PaCa-2 cell surface through an unknown mechanism. Because of the low affinity of the binding, ECUV10c can be detached from Mia PaCa-2 cells while the cells are being washed before fixation. PM: plasma membrane of the Mia PaCa-2 cell. (B) At some point, type I pili can develop binding to mannose residues on the cell surface. The cooperative binding of the original weak adhesion plus pili is stronger and resistant to the washing processes. (C) BSUV9c specifically binds to the Mia PaCa-2 cell surface through an unknown mechanism. Because of their motility and affinity, BSUV9c can attach underneath the cell. (D) The BSUV9c on the surface can be detached by washing, but the BSUV9c under or between cells can remain after washing.

Because *B*. *subtilis* cells are highly motile, movies were captured to see how the interaction between cells and *B*. *subtilis* occurs (S1–S4 Movies show different combinations of bacteria and cells). Interestingly the initial attachments were formed at the leading pole of BSUV9c with the cell surface, implying that the bacterial adhesion molecules accumulate at the pole. These attachments were greatly enhanced for BSUV9c to Mia PaCa-2 cells, relative to all other combinations (Fig 5J). These initial attachments were approximately 5 times more than the bacteria per cell in the adhesion assay following the washing step (Fig 5I), suggesting that many of these bacteria were weakly attached and released by the washing before fixation (Fig 6C and 6D). Interestingly this “weak” attachment actually lasted for more than 4 min once the attachments formed, not only with the combination of BSUV9c and Mia PaCa-2 but also other combinations, i.e., BSUV9c with HPDE cell and wt *B*. *subtilis* with both Mia PaCa-2 and HPDE cells (S3 Fig). This suggests that BSUV9c increases the frequency of forming initial attachment to Mia PaCa-2 cells compared to other combinations.

As described above for *E*. *coli* experiments, the same culture condition for Mia PaCa-2 and HPDE cells were tested to confirm that specific binding of mutant *B*. *subtilis* to Mia PaCa-2 cells is not dependent on the media. Both cells were cultured in Keratinocyte SFM on an FN7-10-coated glass surface, and movies were examined to count the attachment of bacteria. As shown in S5 and S6 Movies the mutant *B*. *subtilis* specifically bind to the Mia PaCa-2 cells in this condition as well. This suggests that the binding of mutant is specific regardless of medium.

### Genomic mutations in BSUV9c

The mutations in the BSUV9c genome were checked by Illumina sequencing (Table 2). First, our original laboratory strain has 26 mutations compared to the 168C sequence in the database (PRJNA57675). Two of these change the amino acid sequences in the genes *uvrX* and *ypqP*. The *uvrX* is reported as a putative UV-damage repair protein in bacteriophage SPβ Although the mutation is a conservative D45E, it might affect survivability after UV treatment. YpqP is an important molecule for biofilm formation in *B*. *subtilis* isolated from hospitals [25]. However, since the expression of *ypqP* is disrupted by bacteriophage SPβ in the strain 168C, this mutation probably does not affect results here.

**Table 2:** Genomic mutations in the lab *B*. *subtilis* 168C strain and BSUV9.

**POS**	**Type**	**168C REF**	**Lab strain 168C REF**	**BSUV** **9c**	**Genes**	**Protein mutation**	**Function**
123468	SNP	T	REF	C	rpoB	I517T	RNA polymerase subunit beta
161239	INDEL	T	TC	TC	rrnI-16S		16S ribosomal RNA
165748	INDEL	TC	T	T	intergenic		
165750	INDEL	TC	T	T	intergenic		
165825	INDEL	G	GC	GC	trnI-Asn		transfer RNA-Asn
166037	INDEL	A	AT	AT	intergenic		
166343	INDEL	AG	A	A	intergenic		
166704	SNP	C	A	A	rrnH-16S		16S ribosomal RNA
171679	INDEL	TA	T	T	rrnG-16S		16S ribosomal RNA
557865	INDEL	G	GT	GT	intergenic		
608214	INDEL	G	GA	GA	intergenic		
651976	SNP	G	REF	A	intergenic		
920823	SNP	C	REF	T	yfiZ	P117L	ABC transporter complex
1026262	SNP	G	REF	A	yhdI	S468F	HTH-type transcriptional regulator
1237380	SNP	C	REF	A	yjbM	Y125STOP	GTP pyrophosphokinase
1261288	SNP	C	REF	T	yjcK	L23L	putative alanine acetyltransferase
1317152	INDEL	G	GGT	GGT	intergenic		
1317153	INDEL	C	CT	CT	intergenic		
1703743	SNP	G	REF	A	fliY	E358K	flagellar motor switch phosphatase
1776900	SNP	C	REF	T	mutS	P386S	DNA mismatch repair protein
1857055	SNP	G	REF	A	pksR	E2056K	polyketide synthase
2097080	INDEL	C	CA	CA	intergenic		
2121679	SNP	C	REF	T	YojG	E210K	scavenge (catabolism)
2151828	SNP	T	A	A	ypqP	F68Y	spore envelope biosynthesis
2271424	SNP	T	C	C	uvrX	R78R	putative UV-damage repair protein
2271505	SNP	C	T	T	uvrX	V51V	putative UV-damage repair protein
2271523	SNP	A	C	C	uvrX	D45E	putative UV-damage repair protein
2480646	SNP	T	A	A	intergenic		
2480647	SNP	A	T	T	intergenic		
2480653	INDEL	CT	C	C	intergenic		
2480666	INDEL	GT	G	G	intergenic		
2581726	INDEL	G	GT	GT	intergenic		
2633673	SNP	G	REF	C	spoIIP	S257STOP	stage II sporulation protein P
2781845	SNP	G	REF	A	yrhE	E213K	formate dehydrogenase
2846024	SNP	G	REF	A	nadA	V346V	quinolinate synthase A
3178443	SNP	T	C	C	rrnB-16S		
3253714	SNP	G	REF	A	comP	Q709STOP	sensor histidine kinase
3256067	INDEL	CT	REF	C	comQ	E281S frameshift	competence regulatory protein
3770058	INDEL	G	GA	GA	intergenic		
3935822	INDEL	AT	A	A	intergenic		
4155390	INDEL	C	CA	CA	intergenic		

POS is the position in the database 168C genome (PRJNA57675). REF is the *B*. *subtilis* 168C sequence. Our lab strain has 26 mutations compared to the database *B*. *subtilis* 168C sequence. BSUV9c has 15 additional mutations.

After 9 cycles of mutation/selection, 15 new mutations were found in BSUV9c. A potential complication is the mutation in the *mutS* gene, which is a mismatch repair enzyme. If the mutation affects MutS activity, the strain may be genetically unstable. Three mutations are in genes for membrane proteins (*comP*, *comQ*, and *yfiZ*), which might affect the cell surface [26]. Several mutations, including *comP*, *comQ*, *yjbM*, *spoIIP*, are in genes known to affect genetic competence and sporulation [27–29].

Another possibility is that the *fliY* mutation might enhance polar attachment because a deletion mutant of FliY does not switch direction of motility [30]. However, the mutation did not enhance the attachment period of BSUV9c on cells, because the profile of duration period for the attachment in each combination of BSUV9c and 168C against HPDE and Mia PaCa-2 cells was essentially the same (S3 Fig).

A clone from BSUV5 (BSUV5c) was investigated as an intermediate strain. This particular clone has mutations in *rpoB*, *yfiZ*, and *yjbM* (S1 Fig and S1 Table). Because these mutations appeared in ~ 40% of the BSUV4 population, which were roughly estimated by the chromatograph for sequencing (S1 Fig), this BSUV5c probably has the character of the BSUV4 population. Although 10% of the BSUV5 population (not a cloned strain) already acquired the *fliY* mutation (S1F Fig) and survived to the next cycles, this cloned strain (BSUV5c) did not acquire the *fliY* mutation and died out by 7^th^ mutation/selection cycle. The affinity level of BSUV5c to Mia PaCa-2 cells is reasonably intermediate between wt and BSUV9c (Fig 5I).

## Discussion

In this study, both Gram-negative *E*. *coli* and Gram-positive *B*.*subtilis* increased their affinity to cancer cells through the mutation/selection process. This bacterial evolution occurred in only 10 cycles of mutation/selection. Several interesting genes were mutated including those for biofilm- and adhesin-related molecules in *E*. *coli*. The mutations in *fimB* (the mutation is 3 bp before the transcription start in the *fimB* promoter P1) and *fimE* are probably major factors for the affinity to Mia Paca-2 cells. FimB and FimE are regulatory recombinases for *fimS*, which is a promoter for type I pili. Both FimB and FimE can recombine and reverse the direction of the *fimS* sequence to suppress the expression of type I pili. This suppression of type I pili is recovered only by the bidirectional recombinase activity of FimE for *fimS*. The mutation in *fimB/E* actually caused a shift to increase the *fimS* direction to on for the *fimA* promoter, which upregulates type I pili expression (Fig 3A, 3B and 3C) Type I pili are known to have affinity to mannose residues and adhere to cell surface receptors. The complete inhibition of ECUV10c adhesion to Mia PaCa-2 cells by mannose, a type I pili inhibitor, suggests that ECUV10c upregulates type I pili expression and thereby enhances adhesion to Mia PaCa-2 cells.

In the case of uropathogenic *E*. *coli*, type I pili adhere to β1/α3 integrin and are involved in the *E*. *coli* invasion of host epithelial cells. Because Mia PaCa-2 cells express β1/α3 integrin more than normal human pancreatic ductal epithelial cells [31], ECUV10c may not only attach more to Mia PaCa-2 cells than HPDE cells but also may be internalized into cells through β1/α3 integrin [32].

However, the specificity for Mia PaCa-2 cells is probably not determined by the type I pili expression level alone, as described in Results (Figs 2 and 3). The ECUV4 population did not have mutations in FimB and FimE genes, but the adhesions to both Mia PaCa-2 and HPDE cells significantly increased compared to the wt W3110. Also ECUV10c, which is supposed to upregulate type I pili expression, showed lower adhesion to HPDE cells compared to ECUV4. To accommodate these obversations I suggest a two-step adhesion process, shown in Fig 6A and 6B.

In this model, the molecule(s) that specifically bind to the Mia PaCa-2 cells developed on the ECUV10c surface by the mutation/selection system. At the first step, ECUV10c may weakly but specifically attach to the Mia PaCa-2 cells through this hypothetical molecule(s) (Fig 6A). Then at the second step, type I pili bind to a cell surface receptor such as β1/α3 integrin Fig 6B). This binding may also be weak, but the cooperative effect of two weak interactions can generate a very strong binding affinity [33]. When mannose inhibits the pilus binding it reduces the affinity to the weak binding of the original molecule. The hypothetical molecule(s) that specifically bind to Mia PaCa-2 cells may be related to biofilm formation.

In order to kill Mia PaCa-2 cells, *S*. *aureus* aHL was expressed in ECUV10c from three different pBAD vectors. The expression level of aHL from the original pBAD18 was not enough to kill Mia PaCa-2 cells. Two new vectors, which were modified from pBAD18, could express aHL in ECUV10c at a level that killed Mia PaCa-2 cells. These results are consistent with previous reports that aHL released by *E*. *coli* after expression from a pBAD vector killed cancer cells. In that study aHL killed breast cancer cells in culture at the same concentration as in the original *E*. *coli* expression media [14]. In the present study, Mia PaCa-2 cells were killed at a 14 times dilution of the aHL in the original expression media. These results suggest that the combination of the strain ECUV10c, pBADMO_aHL and Mia PaCa-2 cells should be optimal both for specific binding to cancer cells and aHL activity. We have not yet tested our strain in mice, but in the previous study bacteria with a similar combination killed cancer cells both in cell culture and in mice [14]. One potential problem in vivo is that the aHL may indiscriminately kill normal cells in the vicinity of cancer cells. Because the protein expression from the pBAD vector is arabinose inducible, a possible solution may be that the amount and timing of administration of arabinose to the model animal are adjusted to right time/level of aHL at the cancer region. Tissue damage by the released aHL in animal models should be monitored.

In the case of *B*. *subtilis*, BSUV9c showed characteristic adhesions on Mia PaCa-2 cells. When BSUV9c swam and hit the surface of Mia PaCa-2 cells at their leading pole, they attached for varied periods and many of them stayed for more than 4 min (S3 Fig and S4 Movie). This distinctive attachment is not strong because the majority of BSUV9c on the top of cells were detached by the washing. BSUV9c attached not only on the top of cells but also underneath cells and intercellular space where BSUV9c remained even after washing. The accumulation of BSUV9c underneath cells and in intercellular spaces is not just caused by motile bacteria sticking in narrow spaces because the wt strain 168C did not accumulate there.

Although the mechanism by which BSUV9c adheres to Mia PaCa-2 cells is not solved, there are several interesting mutations in the BSUV9c genome. Some mutations may change the motility of *B*. *subtilis*. A phosphatase FliY regulates the CheY phosphorylation level which determines the rotational direction of flagella [30]. The deletion of FliY is supposed to make *B*.*subtilis* move forward and reduce the frequency of switching direction. Therefore it is possible that the mutation in the *fliY* gene may inhibit FliY activity and enhance the formation of polar attachment by extending the time for pushing the pole to the cells.

Another line of mutations is in genetic competence and sporulation-related genes including *comP*, *comQ*, *yjbM* and *spoIIP*. *YjbM* is one of the ppGpp synthases and affects both competence and sporulation signaling pathways as well as broad cell systems for the stringent responses [27]. *comP* and *comQ* are mainly involved in the development of genetic competence where ComP phosphorylates master regulator ComA to induce the competence signaling pathway [28, 29]. However, this pathway also communicates with the sporulation pathway [28, 29]. One sporulation specific protein, SpoIIP which is a lytic enzyme on the outside of the plasma membrane, might affect the surface of *B*. *subtilis* [34, 35]. The sporulation and/or genetic competence genes might be involved in the polar attachment although the expression of sporulation genes under normal growth conditions is not known.

A future step is in vivo experiments using animal models, such as a xenograft mouse model using Mia PaCa-2 cells to produce tumors. One should evaluate if bacteria accumulate on cancer cells, if the attached bacteria can kill Mia PaCa-2 cells by induction of toxin, and if the toxin damages normal tissue. ECUV10c is ready to use for the animal model, since a robust toxin expression system was developed here. However, this study is still distant from cancer therapy by two major reasons. First, only one pair of particular pancreatic cancer cell line and normal pancreatic cell line has so far been tested. To further research, additional (pancreatic) cancer cell lines and normal cells should be systematically tested and produce more bacterial strains for many cancer cell lines. Second, the effect of aHL on these additional cell lines should be carefully evaluated. The mutation/selection system developed here may provide an entrée into these more extensive tests and eventual application of bacterial cancer therapy.

## Supporting information

S1 Table Mutation profile in the intermediate strains (ECUV4, BSUV4 and BSUV5c) from mutation/selection. ECUV4 (*E*. *coli* strain after 4 cycles of UV irradiation), BSUV4 (*B*. *subtilis* strain after 4 cycles of UV irradiation) and BSUV5c (a cloned *B*. *subtilis* strain from a single colony after 5 cycles of UV irradiation) were checked for mutations by conventional Sanger sequence methods in the mutant genes that were found in ECUV10c (for ECUV4) or BSUV9c (for BSUV4 and BSUV5c). As expected, ECUV4 and BSUV4 have a mixture of wild type and mutant genes because they comprise a mixture of mutant strains, whereas BSUV5c has a pure genome. Some specific sequence results are shown in S1A–S1F Fig. (a—e) These results showed mixed profiles of wt and mutant. (f). Both BSUV4 and BSUV5c have no mutations in *fliY*. This gene has a mutation in a small percentage of the BSUV5 population, which increased over repetitive selections (S1F Fig).
(PDF)

S1 Fig Mutation profile of mixed populations in the intermediate strains. The mutations that were found in ECUV10c and BSUV9c were checked in intermediate strains. (A-F) correspond to (a-f) in S1 Table. (A) *ycdR* mutation in ECUV3, 4, and 5 were checked. The numbers 3, 4, 5 refer to the cycles of mutation/selection. The mutation increased over repeated mutation/selection. (B) *bglH* mutant population in ECUV4. (C-E) *rpoB*, *yfiZ*, and *yjbM* mutant population in BSUV4. (F) the accumulation of *fliY* mutation population in BSUV4, 5, 6 and 7 over mutation/selection.
(PDF)

S2 Fig Growth inhibition of ECUV10c by the expression of aHL from pBADMOE_aHL. Stationary cultures of ECUV10c transformed with pBADMO_aHL (A, C) or pBADMOE_aHL (B, D) were diluted into induction media containing 0.2% arabinose. Each ECUV10c was diluted 20 (A, B) or 100 (C, D) times and cultured for 2 h. The difference of turbidity was easily observed.
(PDF)

S3 Fig Attachment period of B. subtilis on the cell surface without washing. The attachment periods were measured in movies for each combination. B. subtilis was counted when it stayed the same place on cells more than 2 s. All combinations showed a very similar trend, that is, the majority of B. subtilis detached within 1 min or the attachments lasted for more than 4 min.
(PDF)

S1 Movie BSUV9c added to HPDE cell culture. The interactions with *B*. *subtilis* and cells were captured with movies (DIC). The conditions were the same as adhesion assays described in materials and methods, but prior to washing and fixation. This movie was recorded for 4 min (12x speed).
(MOV)

S2 Movie BSUV9c added to Mia PaCa-2 cell culture. The movie was captured as described in S1 Movie caption. Two asterisks show places where BSUV9c are beneath the cells. This movie was recorded for 4 min (12x speed).
(MOV)

S3 Movie Wild type *B*. *subtilis* added to HPDE cell culture. The movie was captured as described in S1 Movie caption. This movie was recorded for 4 min (12x speed).
(MOV)

S4 Movie Wild type *B*. *subtilis* added to Mia PaCa-2 cell culture. The movie was captured as described in S1 Movie caption. This movie was recorded for 4 min (12x speed).
(MOV)

S5 Movie BSUV9c added to HPDE cell culture with the same condtion as Mia PaCa-2 cell culture in S6 Movie. HPDE and Mia PaCa-2 cells were cultured in the same condition as described in “Methods”. Both cells were cultured on the FN7-10-coated glass surface in Keratinocyte SFM with supplements. This movie was recorded for 4 min (12x speed).
(MOV)

S6 Movie BSUV9c added to MiaPaCa-2 cell culture with the same condition in S5 Movie. This movie was recorded for 3 min (12x speed).
(MOV)
