# Supplementary material for: A repetitive mutation and selection system for bacterial evolution to increase the specific affinity to pancreatic cancer cells
Source: PLoS One. 2018 May 31;13(5):e0198157. doi: 10.1371/journal.pone.0198157 (PMC5979011; doi:10.1371/journal.pone.0198157)
Supplement: S1 Table — ECUV4 (E. coli strain after 4 cycles of UV irradiation), BSUV4 (B. subtilis strain after 4 cycles of UV irradiation) and BSUV5c (a cloned B. subtilis strain from a single colony after 5 cycles of UV irradiation) were checked for mutations by conventional Sanger sequence methods in the mutant genes that were found in ECUV10c (for ECUV4) or BSUV9c (for BSUV4 and BSUV5c). As expected, ECUV4 and BSUV4 have a mixture of wild type and mutant genes because they comprise a mixture of mutant strains, whereas BSUV5c has a pure genome. Some specific sequence results are shown in S1A–S1F Fig. (a—e) These results showed mixed profiles of wt and mutant. (f). Both BSUV4 and BSUV5c have no mutations in fliY. This gene has a mutation in a small percentage of the BSUV5 population, which increased over repetitive selections (S1F Fig). (PDF) [file pone.0198157.s001.pdf]

S1 Table. Mutation profile in the intermediate strains (ECUV4, BSUV4 and BSUV5c) from mutation/selection.

Genes that have a mutation in ECUV10c												
	infA	*cydC*	*ycdR*	*ycfZ*	*yciF*	*ydeH*	*rpoC*	*bglH*	*yjfO*	*yjhT*	*fimB*	*fimE*
ECUV4	wt	wt	mut/wt[(a)]	wt	wt	wt/mut?	mut	wt/mut[(b)]	wt	wt	wt	wt

Genes that have a mutation in BSUV9c										
	rpoB	*ydzT-groEL*	*yfiZ*	*yhdI*	*yjbM*	*fliY*[(f)]	*mutS*	*pksR*	*yojG*	*spoIIP*
BSUV5c	mut	wt	mut	wt	mut	wt	wt	wt	wt	wt
BSUV4	wt/mut[(c)]	wt	wt/mut[(d)]	wt	wt/mut[(e)]	wt	wt	wt	wt	wt

ECUV4 (*E. coli* strain after 4 cycles of UV irradiation), BSUV4 (*B. subtilis* strain after 4 cycles of UV irradiation) and BSUV5c (a cloned *B. subtilis* strain from a single colony after 5 cycles of UV irradiation) were checked for mutations by conventional Sanger sequence methods in the mutant genes that were found in ECUV10c (for ECUV4) or BSUV9c (for BSUV4 and BSUV5c). As expected, ECUV4 and BSUV4 have a mixture of wild type and mutant genes because they comprise a mixture of mutant strains, whereas BSUV5c has a pure genome. Some specific sequence results are shown in S1 Fig (A - F). (a - e) These results showed mixed profiles of wt and mutant. (f) .Both BSUV4 and BSUV5c have no mutations in *fliY*. This gene has a mutation in a small percentage of the BSUV5 population, which increased over repetitive selections (S1 Fig (F)).
