# Supplementary material for: A repetitive mutation and selection system for bacterial evolution to increase the specific affinity to pancreatic cancer cells
Source: PLoS One. 2018 May 31;13(5):e0198157. doi: 10.1371/journal.pone.0198157 (PMC5979011; doi:10.1371/journal.pone.0198157)
Supplement: S1 Fig — The mutations that were found in ECUV10c and BSUV9c were checked in intermediate strains. (A-F) correspond to (a-f) in S1 Table. (A) ycdR mutation in ECUV3, 4, and 5 were checked. The numbers 3, 4, 5 refer to the cycles of mutation/selection. The mutation increased over repeated mutation/selection. (B) bglH mutant population in ECUV4. (C-E) rpoB, yfiZ, and yjbM mutant population in BSUV4. (F) the accumulation of fliY mutation population in BSUV4, 5, 6 and 7 over mutation/selection. (PDF) [file pone.0198157.s002.pdf]

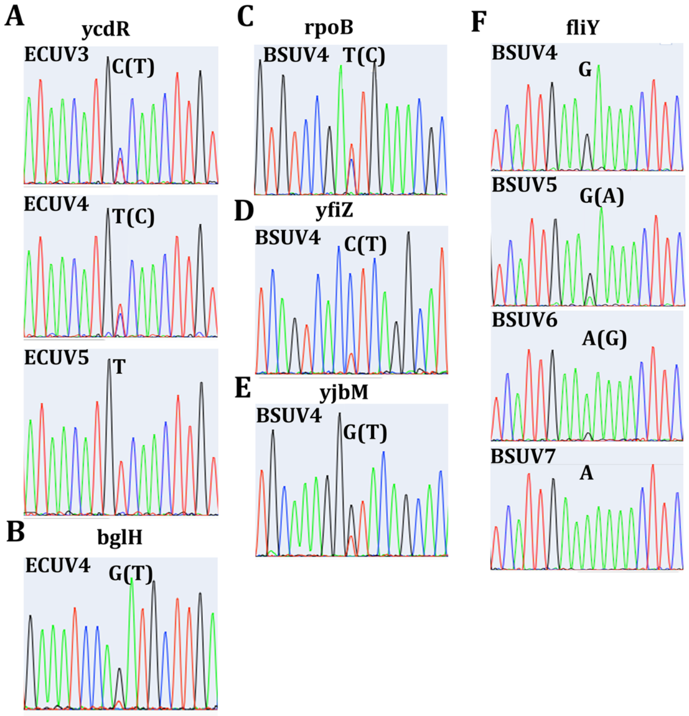

S1 Fig. Mutation profile of mixed populations in the intermediate strains. The mutations that were found in ECUV10c and BSUV9c were checked in intermediate strains. (A-F) correspond to (a-f) in S1 table. (A) *ycdR* mutation in ECUV3, 4, and 5 were checked. The numbers 3, 4, 5 refer to the cycles of mutation/selection. The mutation increased over repeated mutation/selection. (B) *bglH* mutant population in ECUV4. (C-E) *rpoB*, *yfiZ*, and *yjbM* mutant population in BSUV4. (F) the accumulation of *fliY* mutation population in BSUV4, 5, 6 and 7 over mutation/selection.
