# Supplementary figures and images for: A repetitive mutation and selection system for bacterial evolution to increase the specific affinity to pancreatic cancer cells
Source: PLoS One. 2018 May 31;13(5):e0198157. doi: 10.1371/journal.pone.0198157 (PMC5979011; doi:10.1371/journal.pone.0198157)

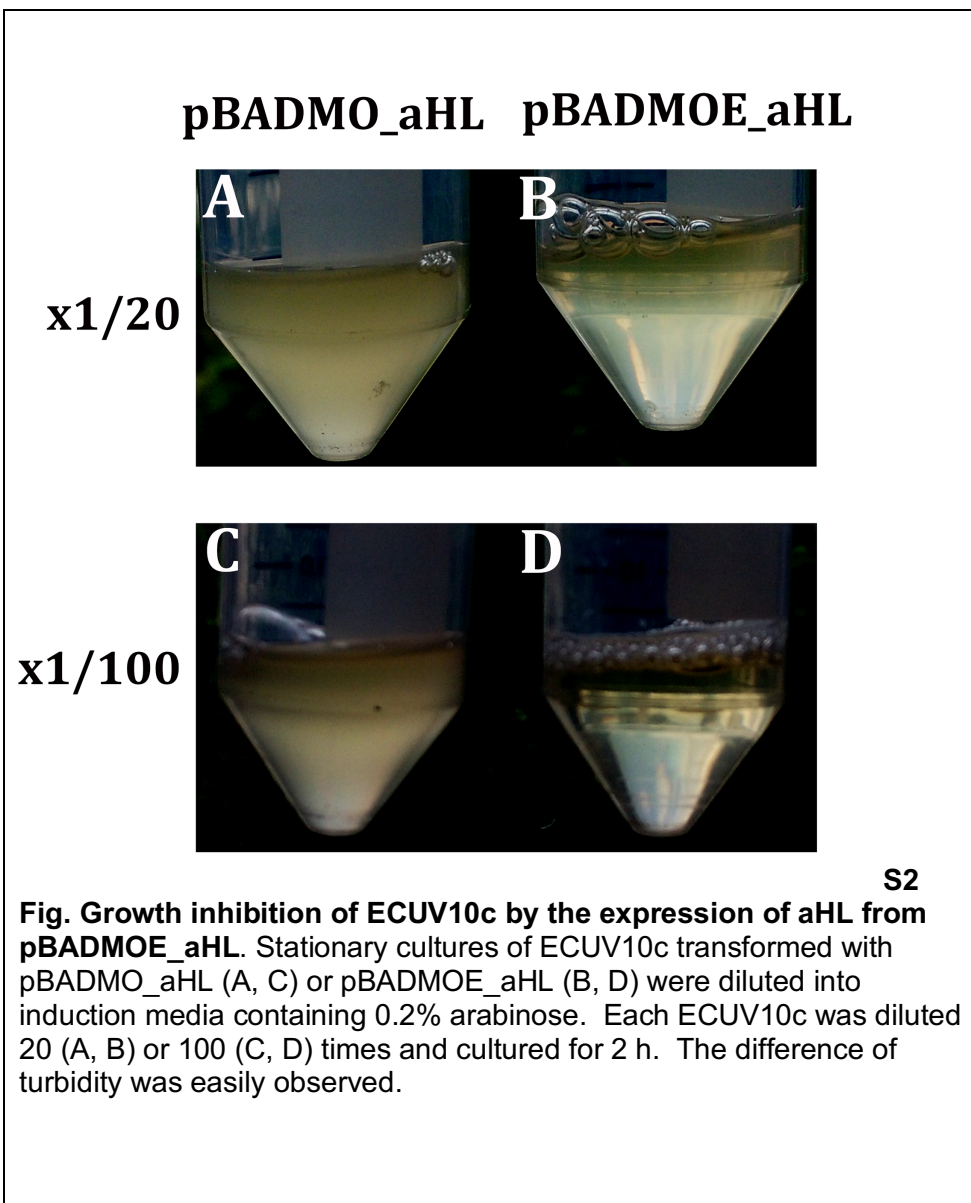

Supplement: S2 Fig — Stationary cultures of ECUV10c transformed with pBADMO_aHL (A, C) or pBADMOE_aHL (B, D) were diluted into induction media containing 0.2% arabinose. Each ECUV10c was diluted 20 (A, B) or 100 (C, D) times and cultured for 2 h. The difference of turbidity was easily observed. (PDF) [file pone.0198157.s003.pdf]
