# Supplementary material for: A repetitive mutation and selection system for bacterial evolution to increase the specific affinity to pancreatic cancer cells
Source: PLoS One. 2018 May 31;13(5):e0198157. doi: 10.1371/journal.pone.0198157 (PMC5979011; doi:10.1371/journal.pone.0198157)
Supplement: S3 Fig — The attachment periods were measured in movies for each combination. B. subtilis was counted when it stayed the same place on cells more than 2 s. All combinations showed a very similar trend, that is, the majority of B. subtilis detached within 1 min or the attachments lasted for more than 4 min. (PDF) [file pone.0198157.s004.pdf]

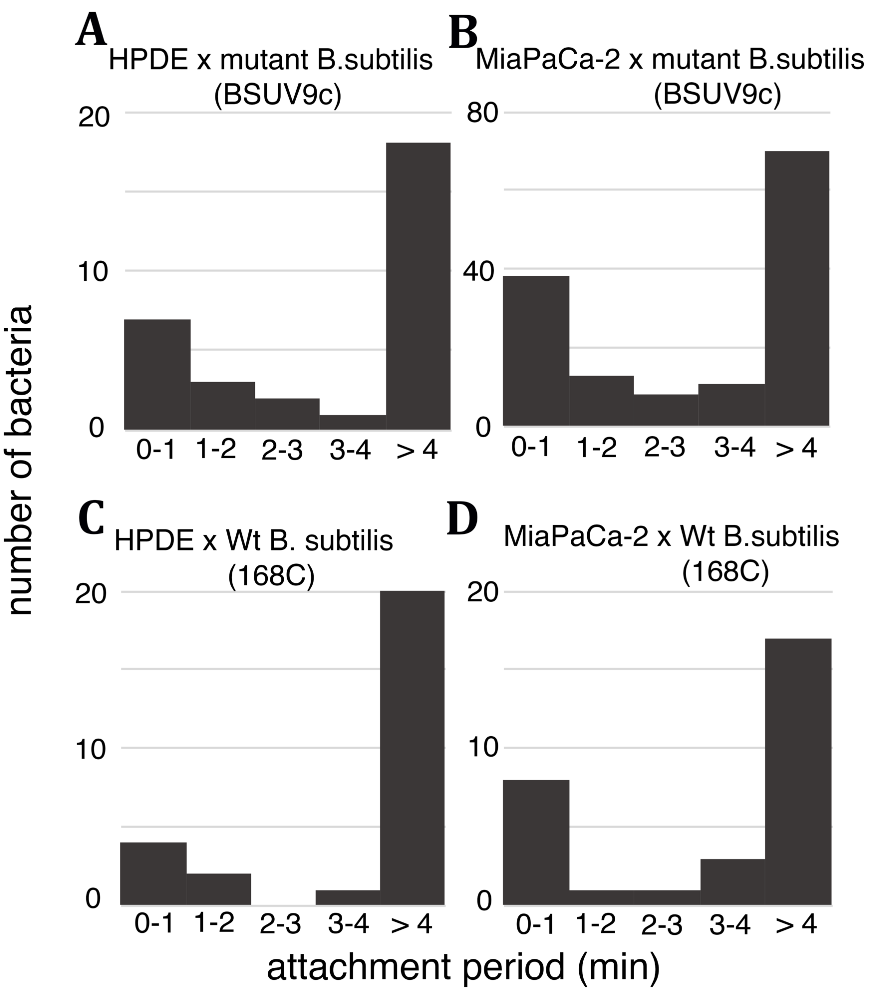

S3 Fig. Attachment period of *B. subtilis* on the cell surface without washing. The attachment periods were measured in movies for each combination. *B. subtilis* was counted when it stayed the same place on cells more than 2 s. All combinations showed a very similar trend, that is, the majority of *B. subtilis* detached within 1 min or the attachments lasted for more than 4 min.
